# Colored Pencil-Core Granuloma With a Long Incubation Period After Penetration: A Case Report

**DOI:** 10.7759/cureus.71887

**Published:** 2024-10-19

**Authors:** Takumi Sakamoto, Msaya Moriwaki, Maiko Tanaka, Akio Tanaka

**Affiliations:** 1 Department of Dermatology, Graduate School of Biomedical and Health Sciences, Hiroshima University, Hiroshima, JPN; 2 Department of Dermatology, Hiroshima Prefectural Hospital, Hiroshima, JPN

**Keywords:** colored pencil-core granuloma, foreign body granuloma, granulomas, green pencil core, infant, pencil-core, skin tumor

## Abstract

Pencil-core granulomas grow rapidly after a long incubation period, typically taking years to decades after penetration by the pencil core. However, colored pencil-core granulomas, which have different core components, have been reported to form just a few days after the core has pierced the skin. It is understood that this difference in incubation period is due to differences in reactivity to different types of foreign bodies. However, we report a rare case of colored pencil-core granuloma that took a three-month incubation period despite being caused by a green pencil-core.

## Introduction

Foreign body granuloma can result from chronic inflammation caused by a foreign body that remains non-degraded in the host body for an extended period.

The duration of granuloma formation differs depending on the foreign body composition. For example, pencil-core granulomas grow rapidly after a long incubation period of years to decades after initial penetration with the pencil [1]. Conversely, colored pencil-core granulomas supposedly form within a few days thereafter. Regular pencil cores are mainly composed of graphite and clay, while a colored pencil core is mainly composed of talc and pigments. These differences in the composition of the core are thought to be the cause of the different reactions, responsible for differences in the time to granuloma formation.

In this report, we present an unusual case of foreign body granuloma caused by a green colored pencil core, which took several months to develop cutaneous symptoms.

## Case presentation

**Figure 1:**
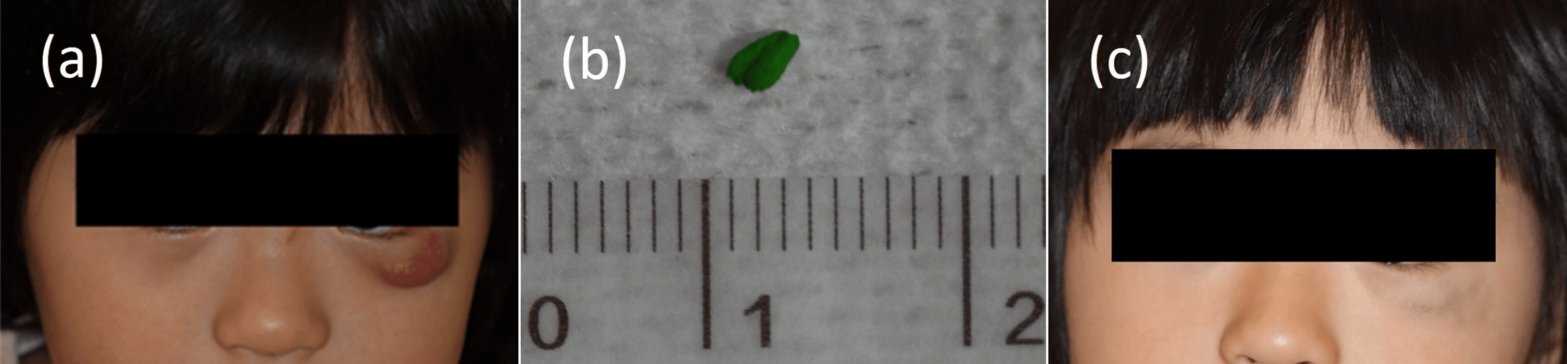
Clinical findings (a) At the first visit, a demarcated red mass was observed on the left lower eyelid. (b) Two months later, a green color pencil core was ejected. (c) Fifteen months after expelling the colored pencil core, the tumor shrank, leaving only a blue pigmentation.

A two-year-old girl presented with a chief complaint of subcutaneous induration of the left lower eyelid that occurred five months before her visit. This was the first visit. At the time of initial diagnosis, she had a 2 cm, clearly demarcated, red, solid tumor on the left lower eyelid (Figure 1a). No obvious signs of trauma were observed on the skin surface, and an ultrasound examination revealed abundant blood flow inside the tumor. The parents were interviewed at the initial visit; there was no history of trauma before the appearance of subcutaneous induration. Despite considering imaging tests, such as CT and MRI, and a pathological skin biopsy, given the requirement of sedation in an infant, the parents decided against it. Based on the clinical findings, she was diagnosed with subcutaneous infantile hemangioma and treated with oral propranolol, but no improvement was observed.

Two months later, the tumor on her left lower eyelid self-destructed, and a 3 mm green pencil core was expelled (Figure 1b). A detailed interview revealed the occurrence of a puncture with a colored pencil on the mucosal side of the left upper lip eight months before her first visit. At that time, the patient visited a local orthopedic surgeon, and no broken core was detected in the wound. The left cheek temporarily swelled after the injury but resolved spontaneously in approximately two weeks. Thereafter, the patient was followed up. The injury occurred in the mouth and it was not recognized as the cause of subcutaneous induration on the cheek. In addition, subcutaneous induration appeared approximately three months after the swelling subsided; thus, the parents may have forgotten about the pencil core.

The tumor then gradually shrank after the removal of the pencil core. Fifteen months after the colored pencil core was removed, only a bluish pigmentation remained (Figure 1c). Because the tumor was on the child’s lower eyelid, no histological examination was performed. However, the clinical course led to the final diagnosis of colored pencil-core granuloma.

## Discussion

Colored pencil-core granulomas are distinguished from pencil-core granulomas by the high permeability of the pencil core. Pencil cores are composed of low-toxicity graphite, silica, and paraffin, and it takes approximately 30 years for the graphite to penetrate the tissue. In contrast, colored pencil cores are composed primarily of talc in addition to titanium dioxide, paraffin, colorants, resins, and additives, which, because of the presence of water-soluble additives, penetrate tissue in a few days. Therefore, colored pencil-core granulomas may cause severe tissue damage within a few days. Currently, only three cases of colored pencil-core granuloma have been reported apart from the present case (Table 1) [2-4]. All these cases involved facial injuries, and the inserted pencil cores varied in color: red, blue, and orange. However, they all caused severe tissue damage within days after injury and required surgical intervention.

**Table 1:** Summary of case reports of colored pencil-core granulomas

Authors	Age of patient	Sex	Location of tumor	Pencil core color	Incubation time	Treatment
Shido and Tamada, 2015 [2]	4 years	Male	Left forehead	Red	Few days	Surgery
Hwang et al., 2023 [3]	18 months	Male	-	Blue	Few days	Surgery
Kaku et al., 2015 [4]	10 months	Male	Right upper eyelid	Orange	Few days	Surgery
Present case	2 years	Female	Left lower eyelid	Green	3 months	Self-destructed

In the present case, the tumor appeared three months after the insertion of the green pencil core, and the incubation period was longer than previously observed with colored pencils. A possible reason for this is the different composition of the colored pencil core compared to previous cases. In experiments on mice in a previous report, yellow, red, blue, pink, brown, and white pencil cores were injected subcutaneously [5]. The results reported that only the yellow and blue colors caused tumors to persist for eight months, while the other colors caused tumors to persist for less than a month. As for the yellow color, it was stated that the pigment Benzidine yellow was likely the cause. We believe that different core compositions respond differently in the body, resulting in large differences in tumor duration. Our case was caused by a different color pencil lead than previously reported, which would explain the different incubation periods. In addition, in our case, the core did not melt 10 months after puncture. The fact that the core remained intact implies a unique course due to differences in its composition. Of course, pencils of the same color may have different compositions that lead to different disease progression.

## Conclusions

We presented a case of colored pencil-core granuloma with a long incubation period. Notably, this feature differed from previous reports. Infants often require sedation for biopsy or imaging, making it more important to complete their medical history via interviews. Given the significant time between injury and mass formation, it was not possible to clarify the patient's injury history with colored pencils during the initial interview. Injuries caused by pencil or colored pencil cores may cause delayed foreign body granulomas, which require a detailed medical history.

## References

[1] Fukunaga Y, Hashimoto I, Nakanishi H, Seike T, Abe Y, Takaku M (2011). Pencil-core granuloma of the face: report of two rare cases. J Plast Reconstr Aesthet Surg.

[2] Shido H, Tamada I (2015). Colored pencil-core granuloma on the forehead. Pediatr Dermatol.

[3] Hwang CJ, Maltry AC, Harrison AR, Mokhtarzadeh A (2023). A case of the blues—colored pencil orbitopathy in an 18-month-old boy. Ophthalmic Plast Reconstr Surg.

[4] Kaku S, Yotani N, Masuda H (2015). Penetrating craniofacial Injury from colored pencil [Article in Japanese]. J Ambul Gen Pediatr.

[5] Matsuo N, Okabe S, Yamamoto K (1972). Eye pelvic granuloma with yellow pencil and its experimental study [Article in Japanese]. Japana Centra Revuo Medicina.

